# Bleeding Risk of Central Venous Catheterization in Adults: A Systematic Review and Meta-analysis

**DOI:** 10.1055/a-2770-0060

**Published:** 2025-12-29

**Authors:** Nicola Mumoli, Lucia Colavolpe, Piero Tarantini, Aldo Fici, Stefania Marengo, Riccardo Capra, Francesco Cei

**Affiliations:** 1 Department of Cardiovascular Medicine, ASST Valle Olona, Ospedale di Circolo, Busto Arsizio (VA), Italy; 2 Department of Internal Medicine, Azienda Ospedaliera, Ordine Mauriziano, Turin, Italy; 3 1st Internal Medicine, Department of Medicine, Azienda USL Toscana Centro, Sede di Empoli, Italy

**Keywords:** CVC, bleeding risk, ultrasound-guided procedures, critically ill patients, blood products transfusions

## Abstract

**Background:**

Central venous catheter (CVC) insertion is a cornerstone procedure in hospitalized and critically ill adults. However, many patients requiring CVCs have coagulopathy, thrombocytopenia, liver disease, or hematologic malignancies, raising concerns about bleeding risk. The true incidence of hemorrhagic complications and the value of preventive measures in these populations remain uncertain.

**Objective:**

The objective of this study is to systematically evaluate the incidence of bleeding related to CVC placement in adults at increased hemorrhagic risk and to assess the effectiveness of periprocedural preventive strategies.

**Methods:**

PubMed, Embase, Cochrane Library, and Web of Science were searched from January 2000 to March 2025. Randomized trials and observational studies involving adults with elevated bleeding risk undergoing CVC placement were included. Data extraction and risk of bias assessment (RoB 2 and Newcastle–Ottawa Scale) were performed independently by two reviewers. Certainty of evidence was rated using GRADE (Grading of Recommendations Assessment, Development, and Evaluation), and random-effects meta-analyses were conducted when appropriate.

**Results:**

Forty-one studies encompassing 7,603 patients and 8,796 CVC insertions were analyzed. Major bleeding occurred in 0.57% of procedures and minor bleeding in 8.1%. The pooled incidence of any bleeding across 22 studies was 6.8% (95% confidence interval, 3.7–10.7%). Bleeding was more frequent among patients with hematologic malignancies, severe thrombocytopenia, or critical illness. Ultrasound guidance markedly reduced complications compared with landmark technique. Platelet transfusion was effective only below 30 × 10 ^9^ /L, whereas fresh-frozen plasma showed no clear benefit.

**Conclusions:**

CVC placement in adults with coagulopathy or thrombocytopenia is generally safe. Ultrasound guidance, restrictive transfusion thresholds, and thromboelastography-guided assessment enhance procedural safety and reduce unnecessary transfusions.

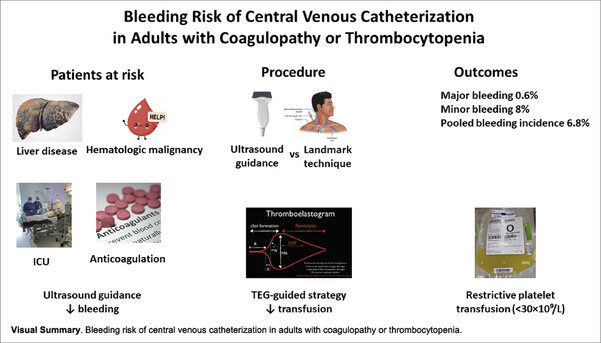

## Introduction

Central venous catheter (CVC) placement is a cornerstone of modern inpatient care. Globally, it is estimated that over 15 million CVCs are inserted annually, with approximately 5 to 8 million procedures in the United States alone. 1 In Europe, the volume is comparably high: for instance, in France and Germany, over 1.5 million CVC insertions per year have been reported in hospitalized adults. 2 These procedures are essential for administering vasoactive agents, intravenous nutrition, chemotherapy, blood products, hemodialysis, and apheresis techniques such as therapeutic plasma exchange (TPE). 3

CVCs can be nontunneled (most common in acute care), tunneled, or implanted ports, with variation in choice based on the expected duration of use and indication. Nontunneled catheters, often inserted into the internal jugular, subclavian, or femoral veins, are preferred in acute and emergency settings due to ease of placement and immediate usability. 4 Tunneled catheters and long-term cuffed lines (e.g., Hickman, Broviac) are primarily used in oncologic patients, chronic dialysis, or long-term apheresis. 5

While the utility of CVCs is undisputed, their insertion is not without risk. Complications include mechanical (e.g., pneumothorax, arterial puncture), infectious (catheter-related bloodstream infections), and hemorrhagic events. Bleeding is particularly concerning in patients with coagulopathy, thrombocytopenia, or those receiving anticoagulant or antiplatelet therapy, all of which are increasingly prevalent in hospitalized and critically ill populations. 6 Reported bleeding complication rates in unselected populations range between 0.5 and 1.6%, with higher risk associated with subclavian site access, lack of ultrasound guidance, and operator inexperience. 7

CVCs are also frequently required in renal replacement therapy, particularly for acute kidney injury in critically ill patients. In such settings, nontunneled large-bore catheters are typically inserted into the right internal jugular vein, considered the optimal site due to favorable flow dynamics and lower complication rates. 8 Similarly, TPE, used in neurologic, hematologic, and rheumatologic emergencies, necessitates large-lumen central access often placed under urgent conditions.

In emergency or critical care environments, the urgency of vascular access frequently outweighs the ability to correct underlying coagulopathies. Given the increasing prevalence of patients with underlying bleeding risk, including those with cirrhosis, hematologic malignancies, or chronic anticoagulation and the frequent need for urgent vascular access, a systematic evaluation of the safety profile of CVC insertion in this population is critical.

The present review aims to synthesize existing evidence on hemorrhagic complications associated with CVC insertion in adult patients at increased risk of bleeding. Furthermore, we explore the efficacy of periprocedural treatments and precautions aimed at reducing the risk of bleeding in many high-risk situations.

## Methods

This systematic review was conducted in accordance with the Preferred Reporting Items for Systematic Reviews and Meta-Analyses guidelines. 9 The research question was structured using the Population, Intervention, Comparison, Outcome (PICO) framework, as follows:

### Population

We included studies involving adult patients (aged > 18 years) at increased risk of bleeding, defined by the presence of at least one of the following conditions: thrombocytopenia; altered coagulation parameters such as prolonged prothrombin time (PT) or activated partial thromboplastin time (aPTT); oral or parenteral anticoagulant therapy; antiplatelet therapy; hepatic failure; hematologic or oncologic disease; or critical illness requiring admission to an intensive care unit (ICU).

### Intervention

We considered studies investigating CVC placement, regardless of the insertion site (jugular, subclavian, axillary, or femoral veins), use of ultrasound guidance, or tunneling. Studies on dialysis catheter placement were also included. Also, peripherally inserted central catheters (PICCs) are considered.

### Comparison

Studies comparing different aspects of CVC insertion were eligible, including: varying bleeding risk profiles, different insertion sites or techniques, and the use or omission of blood product administration to correct coagulopathy. We also included studies focused on rare conditions or descriptive epidemiological analyses that aimed to quantify bleeding risk in high-risk patients undergoing CVC insertion.

### Outcomes

The primary outcomes were the occurrence of major and minor bleeding events following the procedure. Studies in which bleeding was not the primary outcome but was still reported were also included. Secondary outcomes included the need for postprocedural interventions related to bleeding complications.

We included all studies meeting the above PICO criteria that were published between January 2000 and March 2025. To describe the current state of evidence and exclude outdated procedures, we restricted inclusion to studies published in English. To ensure methodological quality and relevance to clinical practice, we searched PubMed, Embase, Cochrane Library, and Web of Science, and we also screened reference lists of relevant guidelines and previous systematic reviews. PubMed indexing was used as an additional quality filter, given that it implies passing the National Library of Medicine's quality assessment. The literature search combined the following Medical Subject Headings (MeSH) and text terms:

(“Central Venous Catheters”[Mesh] OR “central venous catheter”[tiab] OR “central line”[tiab] OR “CVC”[tiab]) AND (“Coagulopathy”[tiab] OR “Blood Coagulation Disorders”[Mesh] OR “Thrombocytopenia”[Mesh] OR “thrombocytopenia”[tiab] OR “coagulopathy”[tiab] OR “coagulation disorder”[tiab] OR “INR”[tiab] OR “aPTT”[tiab] OR “hypofibrinogenemia”[tiab] OR “Anticoagulants”[Mesh] OR “anticoagulant therapy”[tiab] OR “Antiplatelet Therapy”[Mesh] OR “antiplatelet”[tiab] OR “Liver Diseases”[Mesh] OR “hepatic dysfunction”[tiab] OR “cirrhosis”[tiab]).

We included the following types of studies: randomized controlled trials (RCTs), prospective and retrospective analytical observational studies (cohort, case–control, and cross-sectional), and descriptive observational studies (case series with ≥ 10 patients).

We excluded studies meeting any of the following criteria: pediatric populations; lack of extractable data specific to high-risk patients; focus on noncentral venous access devices (e.g., midlines); reports on complications, not including bleeding; single case reports; small case series (<10 patients); studies describing CVCs placed during major surgery in the operating room; and studies without full-text availability; narrative and systematic reviews.

The selection process was independently performed by two reviewers (F.C. and N.M.) using the web-based platform Rayyan (Rayyan, 2025, Cambridge, Massachusetts, United States). All included articles were subsequently reviewed jointly, and any disagreements were resolved through consensus.

The risk of bias was assessed using the RoB 2 tool 10 for randomized studies and the Newcastle–Ottawa Scale (NOS) 11 for observational studies. The overall quality of evidence was rated using the Grading of Recommendations Assessment, Development, and Evaluation (GRADE) approach. 12

Data extracted from the included studies were entered into a standardized, web-based spreadsheet, capturing information on population characteristics, clinical settings, procedural details (site and method of CVC insertion, prophylactic measures), and outcomes (major and minor bleeding events). Extraction was independently reviewed by two authors (F.C. and N.M.).

Major bleedings are defined following the International Society on Thrombosis and Haemostasis (ISTH) criteria 13 :

- Fatal bleeding.
- Bleeding in a critical area or organ (intracranial, intraspinal, intraocular, pericardial, intra-articular, retroperitoneal, intramuscular with compartmental syndrome).
- A decrease in hemoglobin level of at least 2 g/dL.
- Transfusion of at least 2 concentrated blood cell units.

Minor bleedings were all the bleedings, which unmet the previous criteria.

Many studies classified bleeding using the Common Terminology Criteria for Adverse Events (CTCAE), 14 as follows:

- Grade 1: mild, asymptomatic, self-limiting.
- Grade 2: needing minor/local treatment.
- Grade 3: requires major intervention, blood transfusion, or manifests as a drop of at least 2 g/dL of hemoglobin.
- Grade 4: hemorrhagic shock, life-threatening condition, bleeding in a critical area/organ.
- Grade 5: death.

To synthesize the data, we considered major grade 3 to 5 bleedings. Some smaller studies may not adhere to standard definitions of major bleeding and instead use ad hoc criteria. The authors carefully reviewed the case-level definitions and reclassified events, as described in the individual reports, according to the ISTH criteria, in preparation for a pooled analysis.

Given the expected heterogeneity in study designs, populations, and outcomes, and the predominance of observational and qualitative data, we conducted, first, a narrative synthesis. The GRADE approach was used to prioritize and weigh the evidence, with data from studies rated as high or moderate quality considered more reliable. Nonetheless, small observational studies focused on rare conditions were also considered relevant, even if rated as low or very low quality by GRADE.

To enhance the generalizability of the principal outcomes, a pooled analysis was conducted. Pooled analyses were conducted when the considered outcome was explored by at least three studies, so we analyzed three scenarios. First, we synthesized data from studies that primarily reported the overall bleeding rate; second, we examined studies evaluating the impact of platelet transfusion on the bleeding rate; and finally, we assessed the impact of using peripheral venous access. Frequencies and odds ratios (ORs) with 95% confidence intervals (CIs) were calculated using MedCalc Statistical Software (version 23.3.4, 2025; MedCalc Software Ltd). As high heterogeneity was expected, a random-effects model was applied to account for between-study variability in all the analysis conducted, and statistical heterogeneity was assessed using the I ^2^ statistic. Egger's test for publication bias was calculated. Some studies were very small and contained zero events; nevertheless, we included them in the pooled analyses. We did so, first, to account for heterogeneity in bleeding reporting, thereby counterbalancing potential overreporting in other studies, and, second, because selectively excluding such studies would be more distorting, by reducing sample size and precision.

This systematic review was registered in the PROSPERO database [registration number: CRD420251090078].

## Results

**Fig. 1:**
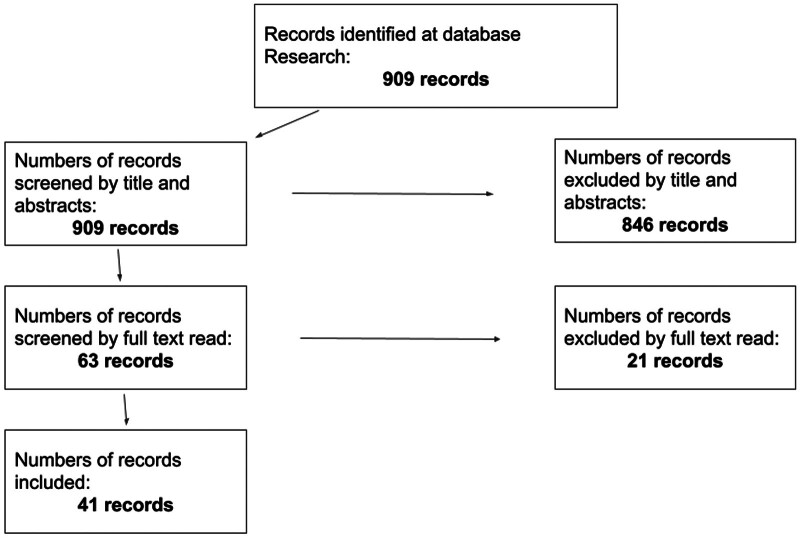
Flowchart for the selection process.

A bibliographic search using MeSH terms yielded 909 articles of which 41 studies met the inclusion criteria and were retained after the selection process detailed in Fig. 1 .

The general characteristics of the included studies are summarized in Table 1 for studies assessing the general bleeding risk in diverse situations and Table 2 for studies assessing specific measures to reduce the bleeding risk. Three were RCTs, while the remaining were observational studies, with 23 having a retrospective design. Among the RCTs, one 15 was judged to have “some concerns” for bias based on the RoB 2 tool ( Table 3 ). Ten observational studies received high scores on the NOS ( Table 4 ). Only two RCTs 16 17 were rated as high quality according to the GRADE framework, whereas one 15 was rated as moderate. Among the observational studies, 8 18 19 20 21 22 23 24 25 received a moderate GRADE rating, whereas the remaining were assessed as low (11 studies) or very low (19 studies) quality ( Table 5 ).

**Table 1:** General characteristics of the studies assessing the overall bleeding rates

Author (y)	Design	Population	Interventions	Main outcome	Results synthesis
van Baarle FLF (2022) 16	Retrospective cohort study	Adult ICU patients with a PLT less than or equal to 50 × 10 ^9^ /L, with or without coagulopathy (INR > 1.5, aPTT > 45 s)	CVC placement (usually except one with echo guidance)	The occurrence of any postprocedural bleeding after CVC placement within 24 h	284 CVC positioned, 44 followed by bleeding, and no significant additional risk for patients with coagulopathy
Napolitano M (2013) 36	Retrospective study	Adult hematologic unit patients with a low PLT	Urgent US-guided CVC placement in patients with PLT < 30 × 10/L vs. patients with count > 30 × 10 ^9^ /L	Major/Minor and late/early complications, including bleeding	431 patients, 39 with severe thrombocytopenia; no major complications, 7 hematomas in the severe thrombocytopenia group
Thompson CA (2009) 53	Retrospective study	Adult patients with AL amyloidosis and factor X-associated deficiency	Invasive procedures, including CVC placement	Bleeding prevalence	25 CVC placements (112 total procedures) with 6/12 bleeding complications
Rockholt MM (2022) 18	Retrospective observational study	Adult patients admitted to the Hematology Department	Echo-guided CVC placement	Mechanical (including bleeding) and infectious complications	89/589 bleeding complications, 61 grade 2–4, more frequent in patients with thrombocytopenia
Theodoro D (2010) 47	Prospective observational study	Patients admitted to the Emergency Department	Echo-guided CVC placement	Mechanical complications, including bleeding	37 complications, 20 hematomas, no differences in patients with an INR > 2
Potet J (2015) 19	Retrospective study	All the patients subjected to CVC placement at the Interventional Radiology Department	PICC echo-guided placement	Procedure-related bleeding	89 patients in antiplatelet therapy, 269 with thrombocytopenia, 42 with PT/aPTT alteration, 23 with DIC, no significant bleeding, 14 hematomas
Rees PSC (2015) 29	Observational study	Patient positive for Ebola virus in Sierra Leone	CVC echo-guided placement	Mechanical complications, including bleeding and bloodstream infection	23 CVC placed, 18 with altered coagulation tests, and 7 with thrombocytopenia, no bleeding was detected
Potet J (2012) 40	Prospective observational study	Cancer (prevalently hematologic diseases) patients with profound thrombocytopenia (< 50 × 10 ^9^ /L)	PICC echo-guided placement	Minor and major bleeding	143 PICC placement, no major hemorrhages, 8 minor hemorrhages, mostly in hematologic patients
Cavanna L (2019) 37	Retrospective study	Cancer patients with a PLT below 20 × 10 ^9^ /L	Echo-guided internal jugular vein CVC placement	Mechanical complications, including bleeding	110 patients, no platelet transfusion, 1 minor bleeding, no major complications
Nasr-Esfahani M (2016) 48	Observational study	Acutely severely ill patients in the emergency department in Iran	Echo-guided CVC placement in patients with or without overt coagulopathy	Major or minor bleeding	59 patients, 35 with coagulopathy, 7 with minor bleeding, no significant increased risk for coagulopathy patients
Erkurt MA (2014) 54	Observational study	Class I HELLP patients (platelet count below 50 × 10 ^9^ /L)	Plasma exchange, CVC placement	Mechanical complications	21 patients, no bleeding
Della Vigna P (2009) 30	Retrospective observational study	Cancer patients with mild coagulation abnormalities	Echo-guided CVC placement	Mechanical complications, including bleeding	157 patients with 239 CVC placements, only 1 minor bleeding
Cortellezzi A (2003) 38	Retrospective observational study	Patients with hematologic malignancies	CVC placement	Mechanical and infective complications	207 CVC placement in 126 patients, 86 with severe thrombocytopenia, no major bleeding
Yeh JH (2001) 55	Observational study	Patients with myasthenia gravis	Plasmapheresis, CVC placement	Postprocedural bleeding, coagulopathy	32 patients, 31 CVC placements, 6 local hematomas, one major bleeding after CVC removal
Weigand K (2009) 28	Prospective observational study	Patients with INR > 1.5 and/or platelet count < 50 × 10 ^9^ /L	Echo-guided CVC placement	Mechanical complications, including bleeding	196 patients, mostly with noncancer conditions, 34 patients with a drop in hemoglobin (7 in those with coagulopathy)
Vinson DR (2014) 20	Retrospective cohort study	Sepsis patients with coagulopathy (INR > 1.3, PLT < 100 × 10 ^9^ /L)	Echo-guided CVC placement	Major and minor bleeding	934 included patients, 204 with combined alterations, 1 major bleeding, 37 minor bleeds, 8 with posthemorrhagic interventions
Wodajo A (2024) 21	Prospective observational study	Adult patients subjected to plasmapheresis	Plasmapheresis, CVC placement, CVC removal in patients with or without a fibrinogen level below 100 mg/dL	Major and minor bleeding	1,406 plasmapheresis in 275 patients, 62 with low fibrinogen levels, only 2 minor bleedings, 152 with CVC insertion
Shah A (2015) 22	Prospective observational study	Adult patients with cirrhosis in India	Major and minor invasive procedures in patients with or without coagulopathy (INR > 1.5 and/or PLT < 50 × 10 ^9^ )	Major and minor bleeding	380 patients, 128 with coagulopathy, 15 combined, 2 patients with clinically relevant bleeding after CVC placement
Haque W (2021) 50	Retrospective observational study	Adults with thrombotic thrombocytopenic purpura	CVC placement	Major and minor bleeding	69 TTP episodes in 61 patients, 9 bleeding, 2 major after femoral insertion
Boban A (2015) 26	Retrospective observational study	Adults with Haemophilia A or B requiring major surgery	CVC placement for blood product transfusion	Insertion-related mechanical complications, including bleeding	65 CVC in 39 patients, no CVC-related bleeding

Abbreviations: aPTT, activated partial thromboplastin time; CVC, central venous catheter; HELLP, Hemolysis, Elevated Liver enzymes and Low Platelets; INR, international normalized ratio; PICC, peripherally inserted central catheter; PLT, platelet; TTP, thrombocytopenic purpura.

**Table 2:** General characteristics of studies addressing specific measures: echo-guide, platelet transfusion, fresh-frozen plasma transfusion, use of peripheral access, and the role of thromboelastometry

Author (y)	Design	Population	Interventions	Main outcome	Results synthesis
Impact of ultrasound					
Karakitsos D (2006) 17	RCT	Adult mechanically ventilated ICU patients	Echo-guided vs. landmark method CVC placement	Successful placement of CVC, mechanical complications (including artery puncture, skin hematoma, hemothorax, pneumothorax, catheter malposition)	The success rate was significantly lower, and complications were significantly higher with the Landmark Method. Hemothorax and hematoma are more frequent with the Landmark methods in patients with and without coagulopathy
Bjorkander M (2018) 27	Retrospective multicenter register study	All the patients > 16 years subjected to CVC placement	Echo-guided CVC placement, Landmark Method	Mechanical complications, including bleeding	892/10,949 patients with coagulopathy, 85 bleeding grade 2–4, increased risk for patients with coagulopathy
Cao M (2024) 31	Retrospective observational study	Patients with acute promyelocytic leukemia during induction therapy	Echo-guided or landmark method CVC placement	Bleeding after placement	95 patients, 39 in the CVC group, no periprocedural bleeding, no differences in blood products use
Role of FFP transfusion/single factors					
Carino GP (2012) 46	Retrospective cohort study	Adult ICU patients	CVC placement (most with the Landmark Method), FFP transfusion	Bleeding after CVC placement	Only one postprocedural bleeding in 287 CVC placements, no benefit for plasma transfusion
Muller M (2015) 15	RCT	All patients admitted to the ICU with an INR between 1.5 and 3 and requiring an invasive procedure	Invasive procedures, including CVC placement, FFP transfusion	Procedure-related bleeding	81 included patients, 58 received CVC placement, and no major bleeding, 13 minor bleeding, no differences
Kwon JO (2016) 32	Retrospective cohort studies	All adults with liver impairment (Child Pugh B or C) admitted to the ICU	Prophylactic use of FFP, PCCs, or rFVIIa before invasive procedures, including CVC placement	Rates of achieving an INR < 1.5, minor and major hemorrhage rates	45 included patients, 23 subjected to CVC, failure of FFP in correcting INR, 33 minor bleeds, 11 major bleeds, no differences between groups
Langley AR (2015) 52	Retrospective observational study	Patients affected by Haemophilia A treated with increasing-dose prophylactic FVIII therapy	CVC placement	Mechanical complications, including bleeding	21 patients required 25 CVCs, 15 before enrollment in the study, and only 1 local hematoma
Role of platelet transfusion					
AlRstum ZA (2018) 42	Retrospective study	Adult cancer patients with a PLT < 50 × 10 ^9^ /L	Echo-guided CVC placement, prophylactic platelet transfusion	Postprocedural bleeding prevalence	10/52 grade 1 bleeding, no major bleeding, no differences in patients subjected to platelet transfusion
Zarama M (2023) 49	Retrospective study	Adult patients with a PLT < 20 × 10 ^9^ /L	Echo-guided CVC placement, prophylactic platelet transfusion	Combined major and minor bleeding	221 patients, 72 received a platelet transfusion, no major bleeding, 79 minor bleeding, no differences between groups
Nosari AM (2008) 39	Prospective observational study	Hematologic patients	CVC placement with Seldinger technique, if platelet or coagulation abnormalities, routine transfusions are applied	Bloodstream infection, mechanical complications, including major bleeding	279 patients, no major bleeding, 5 minor bleedings in severely thrombocytopenic patients
van Baarle FLF(2023) 45	RCT	Hematologic and ICU patients with severe thrombocytopenia (between 10 × 10 ^9^ and 50 × 10 ^9^ /L)	Echo-guided CVC placement with or without prophylactic platelet transfusion	Grade 2–4 periprocedural bleeding	373 CVC placement, 9 vs. 22 bleedings in the treated arm, 4 vs. 8 major bleedings
Haas B (2010) 23	Retrospective observational study	Patients with a PLT < 50 × 10 ^9^ /L or INR > 1.5	CVC placement; transfusion used for PLT < 25 × 10 ^9^ /L or INR > 2	Minor and major bleeding	428 patients with thrombocytopenia, 361 with coagulopathy, 44 both, no bleeding
Duffy SM (2013) 51	Retrospective observational study	Patients with thrombotic thrombocytopenic purpura	Plasma exchange, CVC placement, platelet transfusion	Bleeding complications, other complications	57 CVC placement in 55 patients, no major bleeding, 14 transfused patients, 17 minor bleedings, more frequent for PLT < 30 × 10 ^9^ /L
Zeidler K (2011) 24	Retrospective observational study	Patients with hematologic malignancies and thrombocytopenia	CVC placement, platelet transfusions	Major and minor bleeding	604 CVC placement in 193 patients, 182 grade 1 bleeding, 8 grade 2 in patients with less than20 × 10 ^9^ /L, no major bleeding, 145 transfusions when PLT < 50 × 10 ^9^ /L
Insertion sites differences					
Farina A (2019) 33	Observational study	Critical care patients, most in anticoagulation or antiplatelet therapy	Axillary vs. Jugular CVC echo-guided placement	Procedural time, mechanical complications, including bleeding	35 CVC placement, 67% in full anticoagulation, 59% in antiplatelet therapy, no bleeding.
Glen H (2015) 35	Observational study	ICU patients, mechanically ventilated patients	Echo-guided CVC placement in the axillary vein	Mechanical complications	119 patients, 125 CVC placement, 43 with an INR > 1.5, no bleeding
Jing W (2016) 43	Observational studies	Adult patients with variceal bleeding needing devascularization	Jugular CVC vs. power PICC placement	Mechanical complications	36 patients received PICC, 34 CVC, and no major bleeding, 14 minor bleedings
Role of thromboelastometry					
Lukas P (2018) 34	Retrospective observational study	Sepsis patients needing invasive procedures	Coagulation evaluation with thromboelastometry and/or PT/INR before invasive procedures	Major and minor bleedings	33 CVC placements (76 total invasive procedures), no major bleedings despite elevated PT when thromboelastometry was normal
Kander T (2014) 41	Prospective observational study	Patients with bone marrow failure and a PLT below 50 × 10 ^9^ /L	CVC placement after platelet transfusion	Grade 1–5 bleedings, ROTEM values before and after transfusion	39 patients, only 4 grade one bleedings, no major bleedings, improved ROTEM parameters at 1 and 4 h after transfusion
Pandey CK (2017) 44	Prospective observational study	Child Pugh B and C cirrhotic patients	CVC placement, TEG evaluation	Bleeding requiring treatment, causing increased hospital stay	90 patients, 11 bleeding events requiring blood transfusion, 50 patients with abnormal TEG, and 10 with bleeding, 86 with a PT or platelet abnormality, better predictive value of TEG
Sohail MA (2023) 25	Retrospective cohort studies with a propensity score matching	Adult patients with cirrhosis or acute liver failure	Tunneled CVC placement after TEG-guided transfusions rather than traditional thresholds	Number of blood products used, bleeding complications	89 patients were TEG-guided, 275 traditional-treated patients; there was a significant reduction in blood products in the first group, no differences in major and minor bleedings

Abbreviations: CVC, central venous catheter; FFP, fresh-frozen plasma; ICU, intensive care unit; INR, international normalized ratio; PICC, peripherally inserted central catheter; PLT, platelet count; RCT, randomized controlled trial; ROTEM, rotational thromboelastometry; TEG, thromboelastography.

**Table 3:** Bias evaluation for randomized controlled trials (RoB 2 Tool)

Author (y)	Bias from the randomization process	Bias due to deviations from intended interventions	Bias due to missing outcome data	Bias in the measurement of the outcome	Bias in the selection of reported results	Overall risk of bias
Karakitsos D (2006) 17	Low	Low	Some concerns	Low	Low	Low
Muller MC (2015) 15	Low	Low	Some concerns	Some concerns	Low	Some concerns
van Baarle FLF (2023) 45	Low	Low	Low	Low	Low	Low

**Table 4:** Bias evaluation for observational studies (Newcastle–Ottawa Scale)

Author (y)	Selection	Comparability	Exposition	Overall
van Baarle FLF (2022) 16	2	1	3	Intermediate
Carino GP (2012) 46	2	1	2	Intermediate
Napolitano M (2013) 36	3	1	2	Intermediate
Thompson CA (2010) 53	2	1	1	Intermediate
AlRstum ZA (2019) 42	3	1	3	High
Rockholt MM (2022) 18	2	1	3	Intermediate
Zarama V (2023) 49	3	2	3	High
Theodoro D (2010) 47	2	2	2	Intermediate
Bjorkander M (2019) 27	2	1	2	Intermediate
Potet J (2015) 19	2	1	3	Intermediate
Kwon JO (2016) 32	3	1	3	High
Rees PS (2015) 29	2	1	2	Intermediate
Farina A (2020) 33	2	1	3	Intermediate
Potet J (2013) 40	2	1	2	Intermediate
Nosari AM (2008) 39	2	2	2	Intermediate
Cavanna L (2020) 37	2	1	2	Intermediate
Lukas P (2018) 34	2	1	2s	Intermediate
Langley AR (2015) 52	2	1	2	Intermediate
Kander T (2014) 41	2	1	2	Intermediate
Pandey CK (2017) 44	2	1	2	Intermediate
Nasr-Esfahani M (2016) 48	2	1	2	Intermediate
Haas B (2010) 23	3	1	3	High
Glen H (2015) 35	2	1	2	Intermediate
Cao M (2024) 31	3	1	3	High
Erkurt MA (2015) 54	2	1	2	Intermediate
Della Vigna P (2009) 30	2	1	2	Intermediate
Cortelezzia A (2003) 38	2	1	2	Intermediate
Duffy SM (2013) 51	2	1	2	Intermediate
Zeidler K (2011) 24	3	2	3	High
Yeh JH (2001) 55	2	1	1	Intermediate
Weigand K (2009) 28	2	1	2	Intermediate
Vinson DR (2014) 20	2	2	3	High
Wodajo A (2024) 21	3	2	3	High
Sohail MA (2023) 25	3	2	3	High
Jing W (2016) 43	3	1	2	Intermediate
Shah A (2015) 22	3	1	3	High
Haque W (2021) 50	2	1	2	Intermediate
Boban A (2015) 26	2	1	2	Intermediate

**Table 5:** GRADE quality assessment

Author (y)	Inconsistency	Indirectness	Imprecision	Risk of bias	Selective publication	Large effect	Dose response	Confounders	Overall quality
van Baarle FLF (2022) 16	Not serious	Not serious	Not serious	Serious	Not serious	Not high	Not high	None	Low
Carino GP (2012) 46	Serious	Not serious	Not serious	Very serious	Not serious	Not high	Not high	None	Very low
Napolitano M (2013) 36	Not serious	Not serious	Not serious	Serious	Not serious	High	Not high	None	Low
Thompson CA (2010) 53	Serious	Serious	Serious	Serious	Not serious	Not high	Not high	Present	Very low
AlRstum ZA (2019) 42	Not serious	Not serious	Serious	Not serious	Not serious	Not high	Not high	None	Low
Rockholt MM (2022) 18	Not serious	Not serious	Not serious	Serious	Not serious	High	Not high	None	Moderate
Zarama V (2023) 49	Not serious	Not serious	Not serious	Not serious	Not serious	Not high	Not high	None	Low
Theodoro D (2010) 47	Non serious	Serious	Not serious	Serious	Not serious	Not high	Not high	None	Very low
Bjorkander M (2019) 27	Not serious	Serious	Not serious	Very serious	Not serious	High	Not high	None	Low
Potet J (2015) 19	Non serious	Non serious	Not serious	Serious	Not serious	High	Not high	None	Moderate
Kwon JO (2016)	Serious	Serious	Serious	Not serious	Not serious	Not high	Not high	Present	Very low
Rees PS (2015) 29	Serious	Not serious	Serious	Very serious	Not serious	Not high	Not high	Present	Very low
Farina A (2020) 33	Serious	Not serious	Serious	Not serious	Not serious	Not high	Not high	Not present	Very low
Potet J (2013) 40	Not serious	Not serious	Not serious	Serious	Not serious	Not high	Not high	Not present	Low
Nosari AM (2008) 39	Not serious	Not serious	Not serious	Serious	Not serious	Not high	Not high	None	Low
Cavanna L (2020) 37	Not serious	Not serious	Not serious	Very serious	Not serious	Not high	Not high	None	Very low
Lukas P (2018) 34	Not serious	Serious	Serious	Serious	Not serious	Not high	Not high	None	Very low
Langley AR (2015) 52	Not serious	Not serious	Serious	Very serious	Not serious	Not high	Not high	None	Very low
Kander T (2014) 41	Not serious	Not serious	Serious	Very serious	Not serious	Not high	Not high	None	Very low
Pandey CK (2017) 44	Serious	Not serious	Serious	Serious	Not serious	Not high	Not high	None	Very low
Nasr-Esfahani M (2016) 48	Not serious	Not serious	Serious	Very serious	Not serious	Not high	Not high	None	Very low
Haas B (2010) 23	Serious	Not serious	Not serious	Not serious	Not serious	High	Not high	None	Moderate
Glen H (2015) 35	Serious	Not serious	Not serious	Serious	Not serious	Not high	Not high	None	Very low
Cao M (2024) 31	Not serious	Not serious	Serious	Not serious	Not serious	Not high	Not high	None	Low
Erkurt MA (2015) 54	Not serious	Serious	Serious	Very serious	Not serious	Not high	Not high	None	Low
Della Vigna P (2009) 30	Serious	Not serious	Not serious	Very serious	Not serious	Not high	Not high	None	Very low
Cortelezzia A (2003) 38	Not serious	Not serious	Not serious	Serious	Not serious	Not high	Not high	None	Low
Duffy SM (2013) 51	Not serious	Not serious	Serious	Very serious	Not serious	Not high	High	None	Low
Zeidler K (2011) 24	Not serious	Not serious	Not serious	Not serious	Not serious	Not high	High	None	Moderate
Yeh JH (2001) 55	Not serious	Not serious	Serious	Very serious	Not serious	Not high	Not high	None	Very low
Weigand K (2009) 28	Not serious	Serious	Not serious	Very serious	Not serious	Not high	Not high	None	Very low
Vinson DR (2014) 20	Not serious	Not serious	Not serious	Not serious	Not serious	Not high	High	None	Moderate
Wodajo A (2024) 21	Not serious	Serious	Not serious	Not serious	Not serious	High	Not high	None	Moderate
Sohail MA (2023) 25	Not serious	Not serious	Not serious	Not serious	Not serious	High	Not high	None	Moderate
Jing W (2016) 43	Not serious	Not serious	Serious	Serious	Not serious	Not high	Not high	None	Very low
Shah A (2015) 22	Not serious	Not serious	Not serious	Not serious	Not serious	High	Not high	None	Moderate
Haque W (2021) 50	Not serious	Not serious	Serious	Very low	Not serious	Not high	Not high	None	Very low
Boban A (2015) 26	Serious	Not serious	Serious	Very serious	Not serious	Not high	Not high	None	Very low
Karakitsos D (2006) 17	Not serious	Serious	Not serious	Not serious	Not serious	High	Not high	None	High
Muller MC (2015) 15	Not serious	Not serious	Serious	Serious	Not serious	Not high	Not high	None	Moderate
van Baarle FLF (2023) 45	Not serious	Not serious	Not serious	Not serious	Not serious	High	Not high	None	High

Abbreviations: GRADE, Grading of Recommendations Assessment, Development, and Evaluation.

Notes: Inconsistency is considered serious if the rate of bleeding is very low or very high compared with the randomized controlled trials. Indirectness is considered high when the main outcome is not only bleeding and/or patients without coagulopathy are included. Imprecision is considered when the number of patients included is below 100. The risk of bias is evaluated with the RoB 2 tool or the Newcastle–Ottawa Scale when appropriate. Most studies are safety studies, so adverse outcomes are often reported, and in general, the risk of selective publication is low. A large effect is considered when the population is over 300. As the PICO (Population, Intervention, Comparison, Outcome) question is substantially qualitative, generally, the dose–response effect is absent, except for studies in which more levels of a platelet count are considered. Confounders are found in very selective populations.

In total, the 41 included studies reported on 7,603 patients presenting with at least one of the conditions specified in the PICO question, who underwent 8,796 CVC placements. Major bleeding occurred in 50 cases (0.57%), and minor bleeding in 712 cases (8.1%). Three studies 23 24 26 did not report minor bleeding events. One study 24 reported a high overall bleeding rate; however, most cases were classified as grade 1 bleeding, which was not assessed in other studies, whereas grade 2 bleeding rates were comparable across studies. Higher bleeding rates were observed in studies involving hematologic patients, 18 individuals with severe thrombocytopenia, 27 ICU patients with or without coagulation abnormalities, 28 and patients who had undergone devascularization for variceal bleeding. 22 Several small studies 26 29 30 31 32 33 34 reported no bleeding events. Only one study 35 included more than 100 mechanically ventilated ICU patients without hemorrhagic complications.

A variety of clinical scenarios were examined. Hematologic malignancies were the most frequently studied, both in unselected populations 18 31 36 37 38 39 and in patients selected for coagulopathy or thrombocytopenia. 24 39 40 41 Solid tumors with coagulopathy were also investigated. 42 Liver cirrhosis was also commonly addressed, 22 25 32 43 44 as were critically ill patients in ICU settings, both with 15 16 45 and without 17 35 46 overt coagulopathy. Two studies evaluated patients in the emergency department (ED), 47 48 and two others focused on patients with sepsis. 20 34 Four studies included undifferentiated patients with an elevated PT (international normalized ratio [INR] > 1.5), thrombocytopenia (typically <50 × 10 ^9^ /L), or both. 20 23 27 49 Antithrombotic therapy was addressed in only two studies, 19 33 with only one 33 evaluating patients under full anticoagulation. One study 21 examined patients undergoing plasmapheresis with hypofibrinogenemia. Other small studies investigated rare conditions: two focused on thrombotic thrombocytopenic purpura (TTP) 50 51 and two 26 52 included cases similar to hemophilia. One 53 studied patients with light chain (AL) amyloidosis and factor X deficiency; one 54 evaluated patients undergoing plasmapheresis for HELLP (Hemolysis, Elevated Liver enzymes and Low Platelets) syndrome; and one 55 studied patients with myasthenia gravis. One study 29 addressed patients infected with the Ebola virus in a low-income country.

Among the 11 studies rated as moderate or high quality, data from 3,509 patients (46% of the total cohort) were analyzed, accounting for 4,216 CVC insertions (48% of total procedures). These studies reported 30 major bleeding events (60% of all major bleeding episodes, corresponding to 0.71% of procedures) and 395 minor bleeding events (55% of total, 9.4% of procedures).

**Fig. 2:**
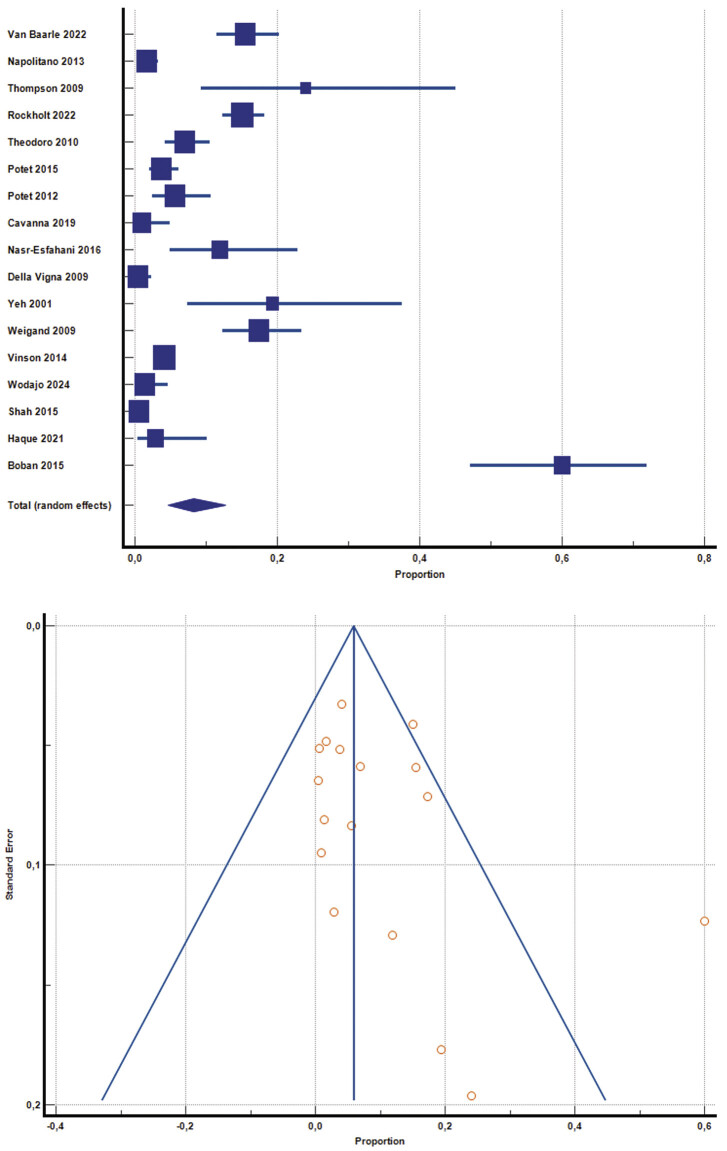
Forest plot of the pooled analysis of 17 studies included for the definition of rate of bleedings after CVC position in patients at increased risk of bleeding (excluding studies with zero events), with relative funnel plot. CVC, central venous catheter (© 2025 MedCalc Software Ltd.).

A pooled analysis was conducted to estimate the overall risk of bleeding in populations at increased risk, based on 20 studies primarily included for the assessment of bleeding rates, as summarized in Table 1 . A total of 4,625 patients with various underlying conditions were evaluated. The pooled random-effects estimate for bleeding risk was 6.8% (95% CI, 3.7–10.7%), with substantial heterogeneity (I ^2^ = 95.2%, *p* < 0.001) (forest plot shown in Fig. 2 ). Egger test negative for publication bias ( *p* = 0.501). To improve the strength of this analysis, we performed a sensitivity analysis, removing the studies with zero adverse events. So, we included 17 studies with 4,374 patients, with an 8.2% (95% CI, 4.7–12.7, I ^2^ = 95.5%, *p* < 0.001) event rate, no publication bias detected ( *p* < 0.001).

One RCT 17 compared ultrasound-guided CVC placement with the landmark technique in mechanically ventilated ICU patients and demonstrated a significant reduction in bleeding and other mechanical complications, even among coagulopathic patients. Most of the included studies employed ultrasound guidance exclusively, although a few also evaluated the landmark technique, 27 31 44 46 with comparable outcomes.

**Fig. 3:**
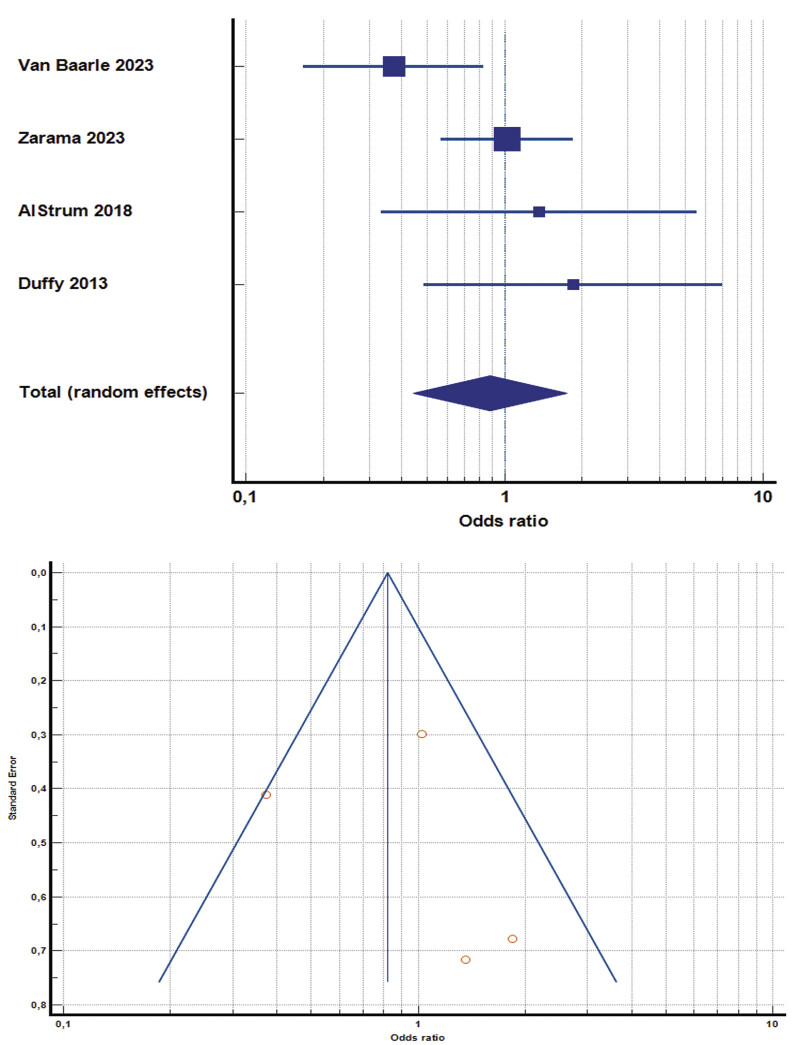
Odds ratio for platelet transfusion, with relative funnel plot (© 2025 MedCalc Software Ltd.).

One study 45 was the only RCT that reported that prophylactic platelet transfusion significantly reduced both major and minor bleeding in hematologic and ICU patients with severe thrombocytopenia, especially when platelet count (PLT) was below 30 × 10 ^9^ /L, despite a small observational study did not find a benefit. The pooled analysis of four studies 42 45 49 51 found a nonsignificant trend for benefit for routine platelet transfusion when PLT falls below 50 × 10 ^9^ cells/L, with an odds ratio of 0.84 (95% CI, 0.46–1.58, *p* = 0.139, I ^2^ = 45.5%). No publication bias detected ( *p* = 0.781) ( Fig. 3 ).

In contrast, one study 15 found no benefit from fresh-frozen plasma (FFP) transfusion in ICU patients with INR values between 1.5 and 3. Another study 41 reported improved thromboelastography (TEG) parameters following platelet transfusion, whereas two others 42 49 did not observe significant benefits. Similarly, three additional studies 32 46 52 found no advantage of FFP transfusion in the setting of elevated INR. One study 25 demonstrated that a TEG-guided transfusion strategy reduced the use of blood products in cirrhotic patients with coagulopathy without increasing bleeding risk. This approach was also supported in sepsis patients, where prolonged K-time (>3.05 minutes) was associated with increased bleeding risk. 44

One study 21 confirmed the safety of CVC placement in patients undergoing plasmapheresis with low fibrinogen levels (<100 mg/dL), regardless of the underlying condition. Similarly, safe CVC insertion was documented in patients with TTP, 51 myasthenia gravis, 55 and HELLP syndrome. 54

**Fig. 4:**
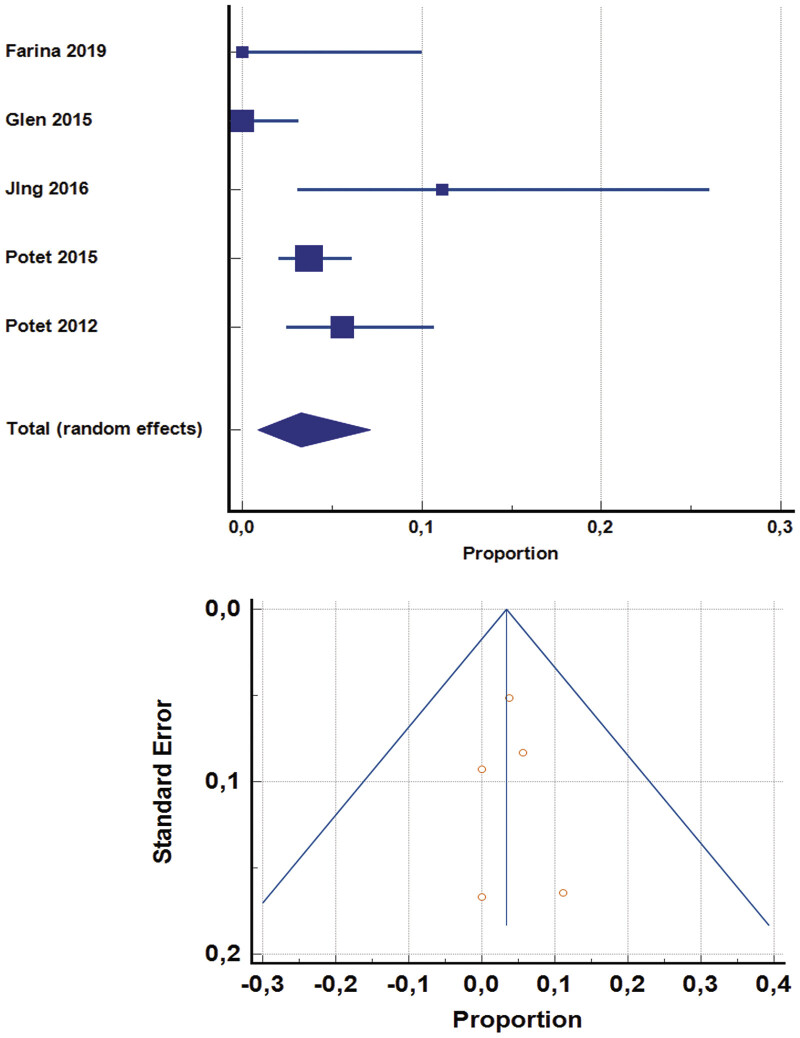
Pooled analysis addressing the expected rate of complications for the peripheral insertion of PICCs in patients with an increased risk of bleeding. PICC, peripherally inserted central catheter (© 2025 MedCalc Software Ltd.).

In 10 studies, the insertion site was not specified, although the internal jugular and subclavian veins were generally favored. Across the remaining studies, the most commonly used access sites were the internal jugular (4,514 insertions) and subclavian veins (1,625 insertions), with no significant differences in bleeding rates. The femoral vein was used safely in 337 cases, whereas the external jugular vein was rarely utilized (43 cases). Three studies 19 40 43 specifically assessed PICCs (557 placements), reporting no major bleeding and few cases of local hematoma (26 cases; 4.7%). Other studies reported an additional 106 PICC placements. Two studies 33 35 examined axillary vein access in 160 cases, all without bleeding complications. Two studies 23 25 addressed tunneled CVCs, which were generally not included in the remaining literature. A pooled analysis of peripherally inserted CVC (including axillary vein and PICCs) was made to estimate the risk of bleeding in these conditions ( Fig. 4 ). Five studies are evaluated with 707 patients, with an estimated risk of 3.3% (95% CI, 0.9–7.1%) with substantial heterogeneity (76.5%, *p* = 0.002). No publication bias detected ( *p* = 0.94). We also performed a sensitivity analysis removing studies with no events; a total of 3 studies with 557 patients and 26 events estimated a risk of 5.5% (95% CI, 2.8–9, I ^2^ = 48.8%, *p* = 0.141). Excluding the two studies determined a significant publication bias ( *p* = 0.003), so we considered the full analysis more accurately. A total of 990 tunneled catheters were placed, with one case of major bleeding (0.1%) and six of minor bleeding (0.6%).

## Discussion

This systematic review included 41 studies, encompassing a total of 7,603 patients and 8,796 CVC insertions. The pooled incidence of major bleeding was 0.57%, whereas minor bleeding events were observed in 8.1% of procedures. Although the overall bleeding rate appears elevated when compared with the general population, 7 the majority of events were classified as grade 1 to 3, indicating limited clinical severity. These findings support the notion that CVC placement remains a generally safe procedure in patients with coagulopathy, provided that appropriate techniques and precautions are employed. Notably, the observed bleeding rate is substantially lower than the 20 to 40% reported in ICU patients with moderate-to-severe thrombocytopenia, 56 and even lower than bleeding risks associated with other invasive procedures such as thoracentesis, where major bleeding has been reported in 1 to 3% of cases. 57 The previous systematic review from van de Weerdt et al. 58 estimated on 22 studies (published before 2015) a prevalence of bleeding between 0 to 32%, which was hard-to-use information in clinical practice. Our quantitative analysis on 4,374 patients found an event rate of 8.2%, most of them minor events. This analysis is weakened by a very high heterogeneity, due to many factors including: many different explored conditions (from sepsis to oncologic patients to cirrhosis and other conditions); differences in defining clinically relevant bleeding; diverse study design (from small case series to RCTs); many different settings (from radiology ambulatory to ED, ICU). However, our study provided a real-world imaging of ambulatories and wars performing CVC placement in many conditions, so we provided a near-real estimate of events in clinical practice. Considering that CVCs are placed in critical or oncologic contexts, the benefit outweighs the bleeding risk, so it can no longer be considered an absolute contraindication to CVC placement. However, all the efforts to minimize the risk of bleeding must be made, including transfusion of blood products when appropriate, insertion performed by full-trained physicians, echo-guided, considering peripheral access, and performing the procedure in adequate structure (ICUs, operating rooms).

A critical observation emerging from this review is the limited predictive value of conventional coagulation parameters, particularly the INR, in assessing procedural bleeding risk. Several included studies demonstrated a poor correlation between elevated INR values and actual bleeding complications during CVC insertion. This is consistent with prior evidence indicating that INR, derived from PT, was designed to monitor warfarin therapy rather than to evaluate hemostatic competence, especially in populations such as patients with cirrhosis. 59 In contrast, TEG appears to offer superior predictive value in critically ill and cirrhotic patients, with studies reporting reduced transfusion requirements and improved risk stratification—even in interventional pulmonology procedures. 60 The use of prophylactic FFP before CVC placement was not associated with a reduction in bleeding events, as evidenced in multiple studies, including one randomized trial. 33 On the contrary, FFP administration may contribute to fluid overload, transfusion-related acute lung injury, resource misuse, and delays in care, particularly concerning patients with heart failure or those requiring ICU-level care. These observations align with the restrictive transfusion strategies recommended by the CHEST guidelines. 61 Thrombocytopenia, frequently considered a contraindication to CVC placement, also appears to be overestimated in its impact. While a PLT threshold of ≥50 × 10 ^9^ /L is traditionally cited, our pooled analysis failed to find a significant benefit (despite a trend being present) in platelet transfusion at that soil. The subgroup analysis of one RCT 16 shows a clearer benefit (even though not statistically significant for the small number of events) when a threshold of 30 × 10 ^9^ /L is considered. This supports a more individualized and risk-based approach, in line with the recommendations of the British Society for Haematology and the last CHEST guidelines, 61 62 considering platelet transfusion only for very severe thrombocytopenia. Importantly, while thrombocytopenia may be more readily correctable through platelet transfusion compared with other coagulopathies, 45 such interventions should be guided by a comprehensive risk–benefit assessment. However, the analysis is largely underpowered, including only four studies with only one RCT with a large sample. To effectively determine the best soil for platelet transfusion across diverse conditions and clarify its true impact, many additional high-quality studies are needed. One of the primary aims of this review was to evaluate the safety of CVC insertion across a spectrum of high-risk clinical conditions. Robust data were available for patients with hematologic malignancies, sepsis, liver cirrhosis, and critical illness—populations frequently presenting with abnormal hemostatic parameters. Despite their high-risk status, the safety profile of CVC placement in these cohorts was favorable. Additionally, data were available for rarer conditions such as TTP, myasthenia gravis, HELLP syndrome, amyloidosis, Ebola virus disease, and hemophilia. However, the evidence supporting CVC use in these populations is derived from low- or very-low-quality studies with small sample sizes. While preliminary data are reassuring, definitive conclusions cannot be drawn, and further investigations are warranted. The advent of real-time ultrasound guidance has revolutionized the safety of invasive procedures, significantly reducing mechanical complications, including hemorrhagic events. This has been well documented in procedures such as thoracentesis 63 and lumbar puncture. 64 In the context of CVC insertion, some studies 17 27 31 44 46 have demonstrated the benefits of ultrasound-guided techniques, particularly in patients with coagulopathy. These findings, adding more recent data, strongly discourage the use of the landmark technique, confirming assumptions derived from older meta-analyses. 65 Although not all studies specified the venous access site, the internal jugular and subclavian veins were the most frequently utilized, with no statistically significant difference in bleeding risk. Femoral vein access, often avoided due to presumed higher bleeding risk, was performed safely in 337 cases. Moreover, our pooled analysis showed a very low risk of bleeding (3.3%) when peripheral veins of the arm (PICC) or axillary veins are used for CVC insertion, suggesting that those options may be suitable in selected populations, such as patients with hematologic malignancies requiring chemotherapy. In cases of difficult jugular or subclavian access, the axillary vein may serve as an alternative, particularly in the critically ill. Tunneled catheters and PICCs were generally safe in coagulopathic patients, although only a minority of studies specifically addressed these devices. Their increasing use in oncology, transplant medicine, and home-care settings warrants consideration of the balance between bleeding, thrombotic, and infectious risks, especially in immunocompromised or septic individuals. Despite the large patient population examined, this review has several limitations. Most included studies were observational (predominantly retrospective and often single center) and thus prone to selection bias, confounding by indication, and outcome misclassification. Definitions of coagulopathy varied widely (e.g., fixed laboratory thresholds, composite clinical criteria, or viscoelastic testing), whereas bleeding severity was graded using disparate systems (ISTH, World Health Organization, BARC, or ad hoc), impairing cross-study comparability. Transfusion thresholds, reversal strategies, and haemostatic assessments were seldom protocolized; minor and procedure-related bleeds were inconsistently ascertained and rarely adjudicated. High-quality randomized trials were scarce; only two studies achieved high certainty of evidence on GRADE, with most downgraded for risk of bias, inconsistency, imprecision, and indirectness. Small sample sizes, single-center designs, and variable follow-up further increased susceptibility to small-study effects and publication bias. We therefore undertook random-effects pooled analyses and complementary sensitivity checks to mitigate these limitations and explore heterogeneity. Nonetheless, substantial between-study heterogeneity persisted across key outcomes, tempering confidence in the summary estimates and underscoring the need for adequately powered, protocol-driven RCTs with harmonized definitions and reporting standards. A limitation of our study is that we restricted inclusion to articles published in English. However, we minimized publication bias by performing a comprehensive search across PubMed, Embase, Cochrane Library, and Web of Science, and by screening references of relevant guidelines and reviews. This strategy ensured that the evidence included represents high-quality, peer-reviewed sources. A particularly notable gap in the current literature pertains to patients receiving anticoagulant therapy, especially direct oral anticoagulants (DOACs). Given the pharmacokinetics of these agents and the absence of reliable monitoring tools such as INR, evidence is urgently needed to inform periprocedural management in this growing patient population. CVC insertion appears to be a safe procedure in patients with coagulopathy or thrombocytopenia, particularly when ultrasound guidance and appropriate clinical precautions are employed. Laboratory abnormalities such as elevated INR or low PLT should not automatically preclude CVC placement, especially in urgent or high-acuity scenarios. Future research should prioritize high-quality RCTs evaluating transfusion strategies guided by viscoelastic assays such as TEG; standardized definitions and reporting frameworks for procedural bleeding outcomes; dedicated studies in underrepresented subgroups, including patients on DOACs and those with rare pathologies. Such efforts are essential to refine risk stratification and optimize procedural safety in this vulnerable patient population.

## Conclusion

We demonstrated that CVC insertion could be considered generally a safe procedure in patients with coagulopathy, including those with thrombocytopenia, elevated INR, cirrhosis, hematologic malignancies, or critical illness. The overall incidence of major bleeding was low (0.57%), particularly when ultrasound guidance was employed, and in most of those critical situations the benefits outweigh the risks.

Traditional laboratory parameters such as INR and PLT show poor predictive value for procedural bleeding risk, and routine prophylactic transfusions—particularly of FFP—do not consistently reduce hemorrhagic complications. Emerging evidence supports the selective use of viscoelastic assays (e.g., TEG or ROTEM [rotational thromboelastometry]) to guide transfusion strategies in high-risk populations, particularly in cirrhotic or septic patients.

Current data support a more individualized, clinically integrated approach to periprocedural hemostatic management, minimizing unnecessary transfusions without compromising patient safety. Nevertheless, the evidence base is largely observational, with significant heterogeneity in bleeding definitions and transfusion thresholds. High-quality randomized trials are needed to define optimal management strategies and procedural protocols in this patient population.

Furthermore, for some conditions (especially patients on oral anticoagulation, very rare diseases), data are very scarce, so the supposed safeness need to be weighed to the lack of specific data.

## Notes

**What is Known About This Topic?**

- Central venous catheterization is frequently required in patients with coagulopathy or thrombocytopenia.
- The bleeding risk in these high-risk populations remains controversial and inconsistently reported.
- Preventive strategies, including transfusions and ultrasound guidance, are variably applied and poorly standardized.

**What Does This Paper Add?**

- Demonstrates that central venous catheter placement is generally safe even in patients with coagulopathy or thrombocytopenia, with a very low incidence of major bleeding.
- Identifies ultrasound guidance and thromboelastography-based assessment as key tools to enhance procedural safety.
- Supports restrictive platelet transfusion thresholds, avoiding unnecessary blood product use.
